# Nano-Electrochemistry and Nano-Electrografting with an Original Combined AFM-SECM

**DOI:** 10.3390/nano3020303

**Published:** 2013-05-17

**Authors:** Achraf Ghorbal, Federico Grisotto, Julienne Charlier, Serge Palacin, Cédric Goyer, Christophe Demaille, Ammar Ben Brahim

**Affiliations:** 1 Applied Thermodynamics Research Unit, National Engineering School of Gabès, Gabès University, Rue Omar Ibn-Elkhattab, 6029 Gabes, Tunisia; E-Mail: ammar.benbrahim@enig.rnu.tn; 2 Laboratory of Chemistry of Surfaces and Interfaces, DSM/IRAMIS/SPCSI, Atomic Energy Commission of Saclay, 91191 Gif-sur-Yvette, France; E-Mails: fedecfedec@gmail.com (F.G.); julienne.charlier@cea.fr (J.C.); serge.palacin@cea.fr (S.P.); 3 Department of Molecular Chemistry, Joseph Fourier University, Grenoble Cedex 09, France; E-Mail: goyerc@gmail.com; 4 Laboratory of Molecular Electrochemistry, Paris VII University, 2 Place Jussieu, Paris Cedex 05, France; E-Mail: demaille@univ-paris-diderot.fr

**Keywords:** AFM, SECM, nano-electrochemistry, nano-electrografting, AFM-SECM, surface, interface, nano-functionalization, nano-process

## Abstract

This study demonstrates the advantages of the combination between atomic force microscopy and scanning electrochemical microscopy. The combined technique can perform nano-electrochemical measurements onto agarose surface and nano-electrografting of non-conducting polymers onto conducting surfaces. This work was achieved by manufacturing an original Atomic Force Microscopy-Scanning ElectroChemical Microscopy (AFM-SECM) electrode. The capabilities of the AFM-SECM-electrode were tested with the nano-electrografting of vinylic monomers initiated by aryl diazonium salts. Nano-electrochemical and technical processes were thoroughly described, so as to allow experiments reproducing. A plausible explanation of chemical and electrochemical mechanisms, leading to the nano-grafting process, was reported. This combined technique represents the first step towards improved nano-processes for the nano-electrografting.

## 1. Introduction

Scanning electrochemical microscopy (referred to as SECM) [1] is one of the major promising tools for surface and interface characterizations. In fact, SECM is often used as a technique to obtain the *in situ* information on a wide range of electrochemical processes occurring at liquid–solid and liquid–liquid interfaces [2]. The spatial resolution of SECM is usually worse than scanning probe microscopy (SPM) techniques, such as atomic force microscopy (referred to as AFM) and scanning tunneling microscopy (STM). Indeed, the improvement of the spatial resolution strongly depends on advances in AFM-SECM combination and electrochemical electrode miniaturization. Therefore, the fabrication of AFM-SECM-electrodes substituting the conventional AFM tips is an interesting and versatile approach combining SECM and AFM. AFM-SECM-electrodes are electrically insulated over all of their surface, but their apex. In fact, AFM gives higher topographical resolution and is able to precisely control the distance between the surface and the apex, whereas SECM can provide detailed information on local anodic or cathodic processes [3,4,5]. Thus, several groups have developed a combined AFM-SECM method, which represents a significant progress in SPM methodology [6]. Recent progress has seen the introduction of new types of SECM-AFM probes, including: (i) microfabricated probes using sophisticated techniques, modifying commercial tips [7,8]; and (ii) homemade probes initiated by the work of Macpherson and Unwin [5,9].

**Scheme 1:**
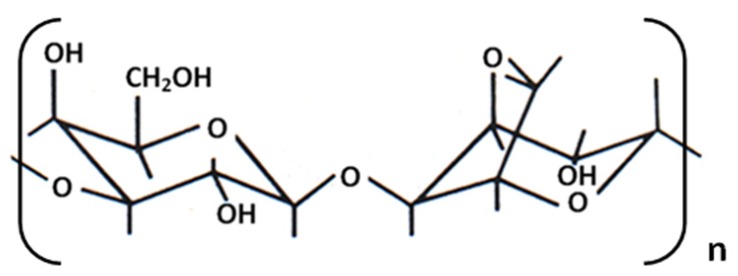
Chemical structure of agarose.

Nevertheless, up to now, work on probes modification or fabrication has essentially been devoted to the analysis of local electrochemical phenomena, particularly for studying biological systems [10,11,12,13]. In fact, the potential of a local conducting probe to electrochemically induce nano-electrografting of non-conducting organic molecules onto conducting surfaces has not been well explored. Indeed, cathodic electrografting is a powerful technique for modifying and patterning conducting surfaces with organomolecules [14,15]. It finds application in various fields, including biocompatibility, sensors, protection again corrosion, lubrication, soldering, functionalization, adhesion, template chemistry [16] and industrial efﬂuent clean-up [17]. Cathodic electrografting of polymers [18] is based on the initiation, then polymerization, of electroactive monomers on the electrode, either conducting or semi-conducting. The adherence of the electrografted films, which can be considered as disordered polymer brushes [19], is ensured by a carbon-metal covalent bond [20]. Cathodic electrografting may proceed via a purely anionic mechanism, under drastic anhydrous conditions, but also via electro-induced radical polymerization (EIRP). Indeed, using diazonium salts as initiators, it is possible to graft vinylic polymer chains to various conducting surfaces in aqueous solutions [21] or micellar suspensions [22]. In the present study, we report for the first time the electrochemical use of uncoated PtIr-AFM probes in aqueous medium using a hydrogel as an electrochemical cell. This interest about agarose (Scheme 1) is related to new and wide applications of hydrogels used as chemical reactors, micro-reactors and electrochemical/photochemical sensors [23,24,25].

Moreover, there is a lack of information about the technical reproducing of AFM-SECM experiments, which limit the expansion of this promising technique. A unique feature of this paper is that, although there are numerous publications dealing with AFM-SECM combination, few of them insist on the experimental difficulties and details related to the combined technique itself. Thus, here, we not only report mechanisms of the nano-electrografting of organic coatings on a homogeneous substrate and prove the possibility of nano-electrochemical processes onto agar surfaces with the original AFM-SECM combination, but also we insist on experimental precautions related to the electrochemical experiment and probes fabrication. The purpose of this approach is the democratization and the improve of this nano-electrografting technique.

## 2. Experimental Section

### 2.1. Hydrogel-Cell Preparation

Three weight-percent (*w*:*w*) of agarose powder were slowly scattered in de-ionized water under stirring to prevent clumping. Water evaporation was prevented by a paraffin paper. The beaker was then heated with a boiling water bath for 5–10 min under continuous stirring, until the agarose dissolved completely. Heating was stopped at 90 °C, and the mixture was allowed to cool. When the temperature had fallen to 65 °C, the solution was poured into a pre-warmed beaker. A tight and elastic gel was then obtained. After cooling, the gel was gently removed from the master, sliced and immersed in the electrochemical solution. The hydrogels were then dipped into the 5 mM ferricyanure and 0.1 M KCl aqueous solution under argon bubbling for 1 h. The gels were quickly dried under absorbent paper before the electrochemical test. A recent study showed that changing the time of diffusion between 30 min and 3 h has no effect on the electrochemical phenomena [26].

### 2.2. Sharp AFM-SECM-Electrode Fabrication and Characterizations

The AFM-SECM-electrode fabrication was entirely based on the Demaille [27] method and advice.

#### 2.2.1. Tip Etching

Homemade probes are produced by electrochemical etching of commercial (gold 99.99%; Goodfellow, Huntingdon, UK) microwires (60 µm in diameter). Before etching, the 1.5 mm length of the gold wire was bent at a right angle, whereas the long extremity of the wire was flattened between two stainless steel plates. The 1.5 mm length wire extremity was then etched electrochemically to form a sharp conical tip. Electrochemical etching was carried out by immersing the wire in a saturated (CaCl:Milli-Q purified water:ethanol) solution (10:40:5; *v*:*v*:*v*) deposited on an aluminum foil. A voltage of +5 V was applied to the gold wire, while the aluminum foil served as the counter electrode. Etching time was complete in about 1 min. The etching solution was renewed every time a new probe was etched.

#### 2.2.2. Tip Insulation

The gold microwire, bearing the preformed sphere at its tip, is immersed in a solution containing cathodic electrophoretic paint (BASF, Cathodip TM FT83-0250) diluted in a 1:1 (*v*:*v*) ratio by water and acidified by 3 mM acetic acid. The gold microwire is then connected to a potentiostat as the cathode of a two-electrode configuration, the anode being a platinum coil. The potential of the microwire is scanned from 0 to −5 V at a rate of 50 mV s^−1^ to trigger deposition of the paint. At such a negative potential, protons are reduced, and the resulting local pH rise causes the deprotonation of the NH^3+^ moieties borne by the polymer chains composing the cathodic paint that then precipitate onto the gold microwire surface. After deposition, the gold microwire is rinsed with water and placed in an oven heated to 180 °C for 20 min. This thermal curing step results in the crosslinking of the amine-bearing chains by diisocyanate linkers also present in the paint composition. When the painted gold microwires were characterized in an aqueous solution of ferrocenedimethanol by cyclic voltammetry, no faradaic current was recorded. This behavior demonstrates that the cured paint layer forms a perfectly insulating coating over the entire surface of the gold microwire.

#### 2.2.3. Selective Exposure of the Sharp Apex of the Microwire

**Figure 1:**
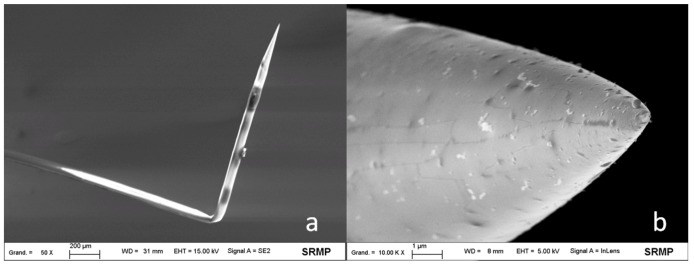
(**a**) Scanning electron microscopy image of the homemade AFM-SECM-electrode after insulation with electrophoretic paint. Beam energy was 15 kV and working distance 31 mm; (**b**) Scanning electron microscopy image of the tip apex. The apex radius was around 130 nm. Beam energy was 5 kV and working distance 8 mm.

A high-voltage pulse, generated by the single pulse/spark generator developed by Demaille [27], was applied to the probe, shown in Figure 1a, to selectively expose its apex-end. The value of the conical tip radius was determined by scanning electron microscopy for several probes and was found between 60 and 150 nm, depending on the probe (Figure 1b).

**Figure 2:**
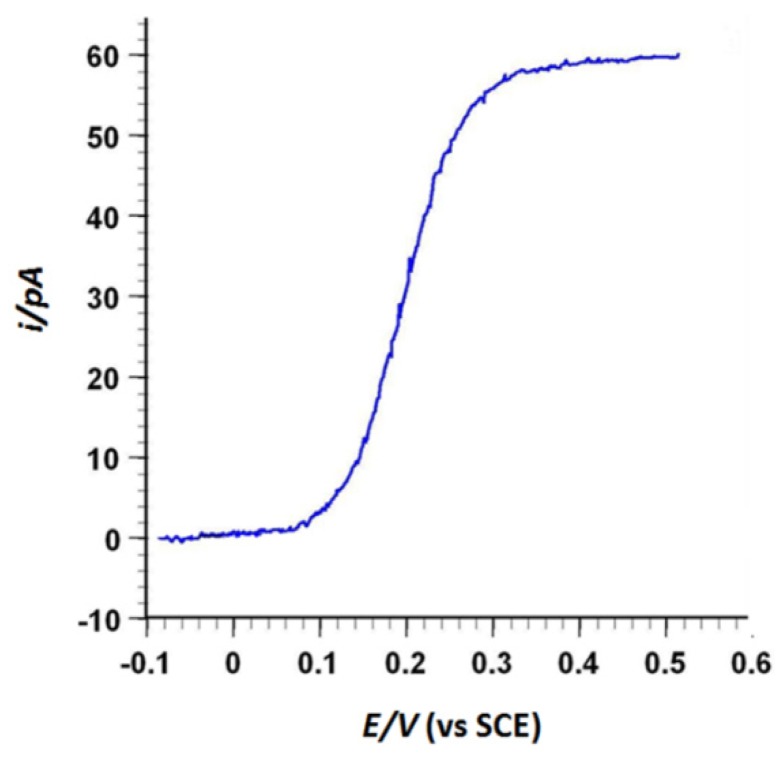
Steady-state voltammogram of the AFM-SECM-electrode in aqueous solution containing 1 mM ferrocenedimethanol and 0.1 M KH_2_PO_4_. The forward and backward traces are superimposed. The potential scan rate was 50 mV s^−1^.

This AFM-SECM-electrode was then characterized by steady-state voltamperometry to get an estimate of the electro-active area of the tip from the limiting current. The well-characterized rapid electron transfer ferrocenedimethanol redox species was used. The typical current-potential sigmoidal curve obtained with an AFM-SECM-electrode is shown in Figure 2.

An interesting aspect of the recorded cyclic voltammetric response is in the absence of hysteresis between the forward and backward traces of the voltammograms. This curved shape is indicative of the relatively low capacitance of the AFM-SECM-electrodes, resulting from the good insulating property of the reticulated paint film. By assuming that the exposed portion of the AFM-SECM-electrode is hemispherical, the diffusion-limited current is expected to follow [1]:

*i*_lim_ = 2 π *n F D C a* (1)

where *n* is the number of electrons involved in the electrochemical reaction, *F* is Faraday’s constant (96,485 C mol^−1^), *D* is the diffusion coefficient of the reacting species (for ferrocenedimethanol: *D* = 7 × 10^−6^ cm^2^ s^−1^) [28], *C* the bulk concentration of the species (10^−6^ mol L^−1^) and “*a*” the radius of the hemisphere. Using this equation, the electrode radius of our AFM-SECM tip was found close to 130 nm.

#### 2.2.4. AFM-SECM-Electrode Electrical Connection

The probe was then glued to a standard (AFM) sized glass chip using epoxy glue (Quick Set Epoxy; Radiospares, Beauvais, France). The back extremity of the probe, expanded from the glass chip, was electrically connected to a thin insulated wire using silver epoxy. This junction was then insulated using a drop of epoxy glue. The probe dimensions were measured by optical microscopy.

#### 2.2.5. AFM-SECM-Electrode Normal Spring Constant Calculation

The normal spring constant (*K*) was estimated using the Sader [29] equation for rectangular cantilevers:

*K* = 0.1906 ρ *b*^2^*Q L* Γ(ω) ω^2^ (2)

where ρ is the density of the cantilever environment. *b*, *L*, *Q* and ω are, width, length, quality factor and the fundamental mode resonant frequency of the cantilever, respectively. Γ(ω) is a function that only depends on the Reynolds number (functionally independent of the cantilever thickness and density). The value of the spring constant, for several probes, was found between 1.5 and 3.5 N/m.

### 2.3. AFM-SECM Experiments Details

The electrochemical experiments were performed with a Molecular Imaging PicoSPMLe AFM microscope (PicoScan 2100 Controller; Scientec, Les Ulis, France). All potentials reported herein are with respect to the silver wire quasi-reference electrode, unless otherwise stated. Moreover, it is important to notice that some important precautions were taken, so as to ensure no tilting drift during electrochemical experiments. The most important precaution was to thermally stabilize the actuator and the cantilever before experiment launch. In fact, as proven by Haugstad and Gladfelter [30], we observed that without thermal stabilization, the actuator shows a hysteresis and nonlinearity in vertical and horizontal displacements. Therefore, we followed a protocol, including 3 h of continual scanning, before the experiment series, so as to consider the lead zirconate titanate (PZT) actuator as thermally stable.

#### 2.3.1. AFM-SECM/Agarose Electrochemical System Validation Experiment

**Figure 3:**
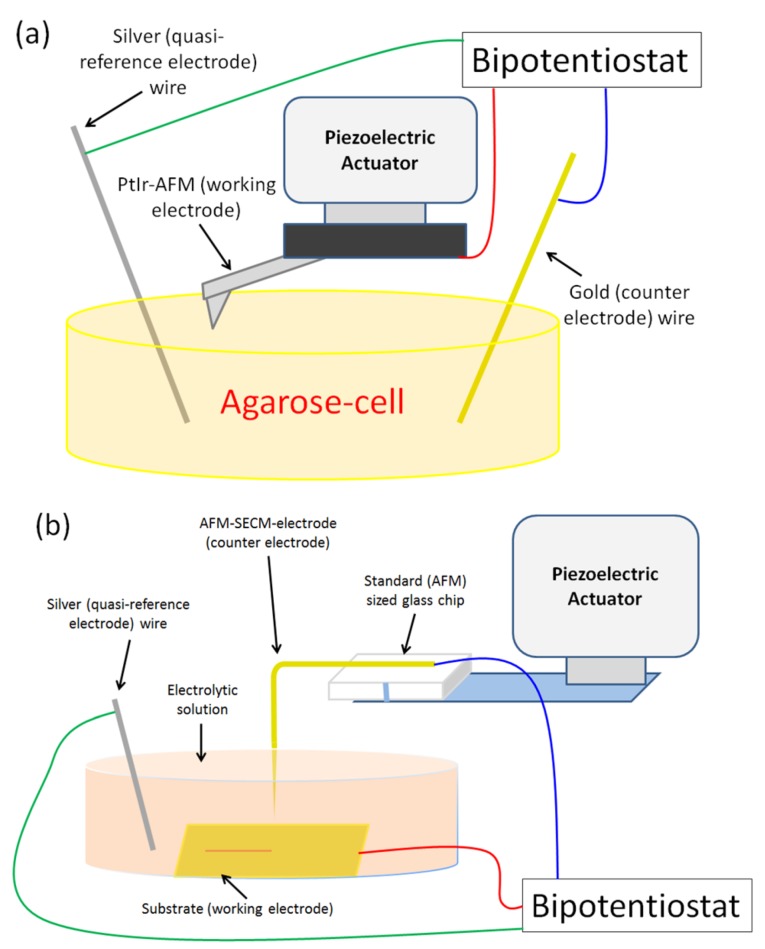
Schematic drawing of AFM setups (**a**) for the AFM-SECM/Agarose system; (**b**) for the nano-electrografting system.

The electrochemical system validation was carried out with the hydrogel as an electrochemical cell. The gel was introduced in the AFM support cell, where silver (quasi-reference electrode) and gold (counter electrode) wires were planted in the agarose (see Figure 3a). A contact mode PtIr-AFM conductive probe (NanoWorld, Neuchâtel, Switzerland) was electrically connected via the metallic probe support holder and used as a working electrode. A classical tip-surface approach was carried out.

#### 2.3.2. Nano-Electrografting Experiment

As shown in Figure 3b, the fabricated AFM-SECM-electrode was used as the counter electrode of a standard three-electrode arrangement. It is important to remember that these fabricated AFM-SECM probes integrate tips of about 1.5 mm length. It is then possible to immerse only the final end of the tip (~200 µm) in the electrolytic solution (300 µL). The substrate (Pyrex glass plates coated with 2 nm of chromium and 100 nm of gold (99.99%) by vacuum evaporation) was the working electrode and a silver wire the quasi-reference electrode. The experiments were performed by adding to the AFM a home-modified fluid cell containing the electrolytic solution (commercial acrylic acid (Aldrich, 0.7 mol dm^−3^) in acidic aqueous solution (H_2_SO_4_ 0.25 mol dm^−3^, analytical grade) in the presence of 4-nitrobenzenediazonium tetrafluoroborate (Aldrich, 2 mmol dm^−3^). Solutions for electrochemistry were not degassed prior to use, and all measurements were made at room temperature. The potentials applied to the electrodes were controlled by an external bipotentiostat (BioLogic Science Instruments). The microscope head was placed inside a Faraday cage in order to block external static and non-static electric fields and mounted on a floating table to achieve vibration isolation during investigations and characterizations.

## 3. Results and Discussion

**Figure 4:**
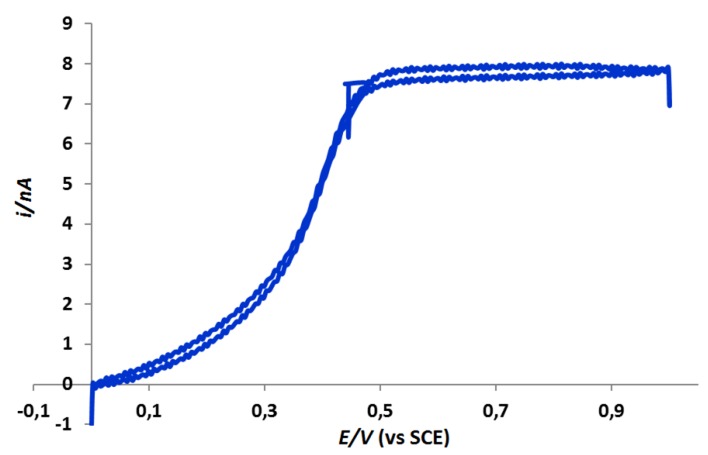
Steady-state voltammogram of the PtIr-tip in contact with the agarose surface. Agarose was previously immersed in aqueous solution containing 5 mM ferricyanure and 0.1 M KCl, during 1 h. The potential scan rate was 50 mV s^−1^.

The AFM-SECM/Agarose system functioning was characterized by steady-state voltamperometry. The well-characterized rapid electron transfer ferricyanure redox species was used. A typical current-potential sigmoidal curve was obtained with an uncoated PtIr-AFM (used to act as an SECM electrode) and is shown in Figure 4.

We have developed a simple, affordable, accessible and quick method for functional nanoscale electrochemical local characterization. This method is based on a simple system composed by an uncoated PtIr-AFM probe and an agarose-cell. The recorded currents in these AFM-SECM experiments were in an easily measurable range of ≈ 8 nA, whereas this measurable range was of about ≈ 12 nA for more complicated systems using microfabricated AFM-SECM probes [31]. In the future, this technique could be extended to many measurements in the physical and life sciences, like quantification of the surface diffusion of binding motifs on agarose surfaces [32].

**Figure 5:**
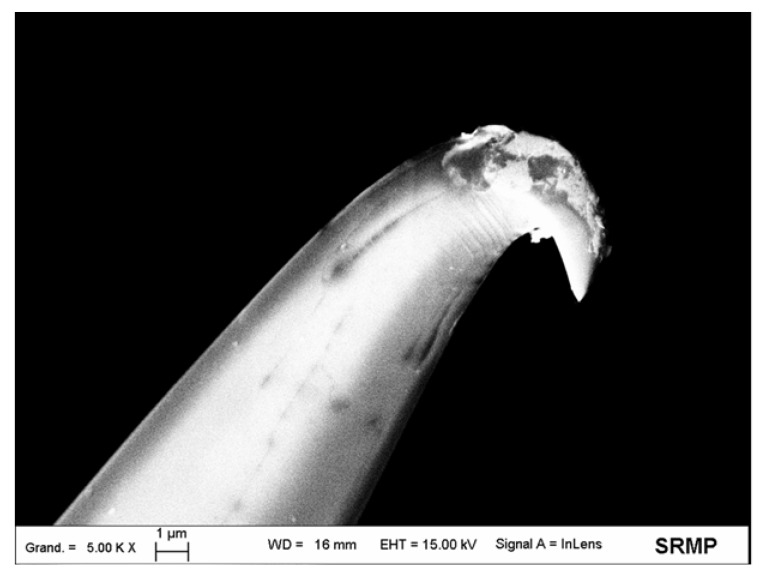
Scanning electron microscopy image of the homemade AFM-SECM-electrode after collision with the surface during nano-electrochemical grafting attempts. Beam energy was 15 kV and working distance 16 mm.

The same AFM-SECM technique was used for nano-electrografting experiments by replacing the PtIr-probe with the above-described homemade AFM-SECM-electrode. The working distance was set by positioning at first the tip “in contact” with the surface and then by withdrawing from the substrate. A 5 µm working distance and a low scan velocity of about (18 µm s^−1^) were found to be optimal to allow nano-electrografting. For smaller distances, the probe becomes too sensitive to the disorder of the solution caused by the gases release, and for larger distances, the localization is lost. Moreover, in this case, the probability of tip-surface collision is very important and often leads to AFM-SECM-electrode damage (see Figure 5).

**Figure 6:**
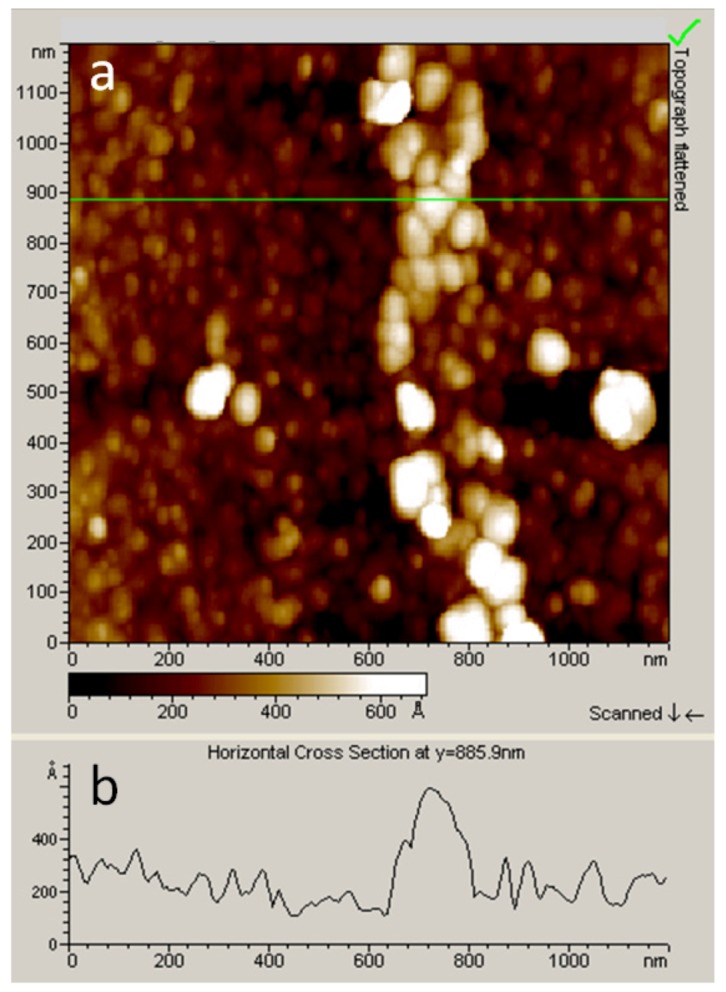
(**a**) Topographic AFM image in tapping mode of the line pattern drawn with the AFM-SECM-electrode on the gold surface with direct aryl diazonium salt/acrylic acid reduction (chronoamperometry: −0.8 V); (**b**) Horizontal cross section of the electrografted line. Line width and height are about 180 nm and 40 nm, respectively.

The electro-initiated polymerization technique was thoroughly described in a previous paper [14]. Briefly, it consists in applying a chronoamperometric technique by setting the potential to −0.8 V *vs*. Ag wire. All the samples were sonicated in ethanol and water before analyses. During AFM-SECM experiments, the tip movement was monitored via the reflection of the AFM laser onto the cantilever arm of the probe and the detection of the reflected beam on a position-sensitive detector. The resulting localized thin films were characterized by topographical AFM analysis (Figure 6). For imaging the localized electrografted trace by AFM in tapping mode, the AFM-SECM-electrode was replaced by a commercial pyramidal Si-tip (mounted on a 225 µm-long single beam cantilever with a resonance frequency of approximately 75 kHz and a spring constant of about 3 N m^−1^). AFM images were recorded using the retrace signal. The scan rate was in the range of 0.25 Hz, with a scanning density of 512 lines/frame.

**Figure 7:**
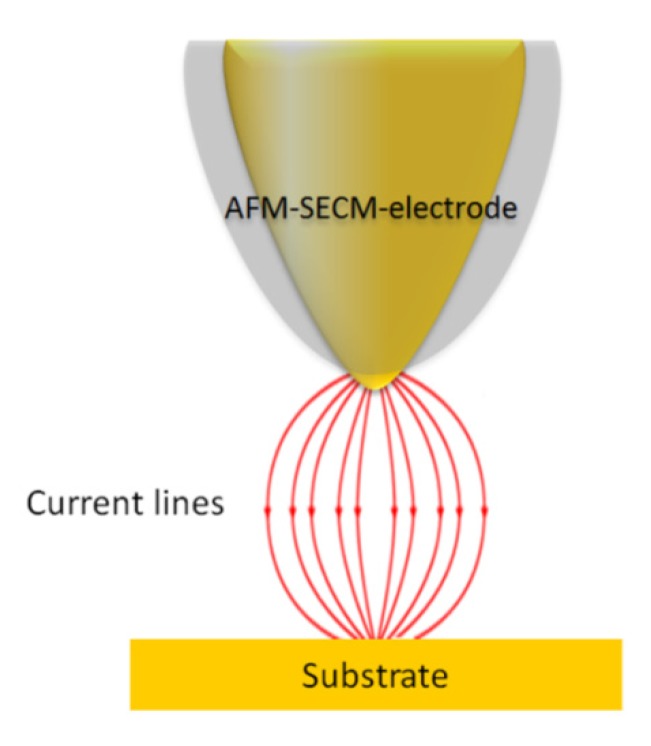
Schematic representation of the electric field between the AFM-SECM-electrode and the substrate.

**Figure 8:**
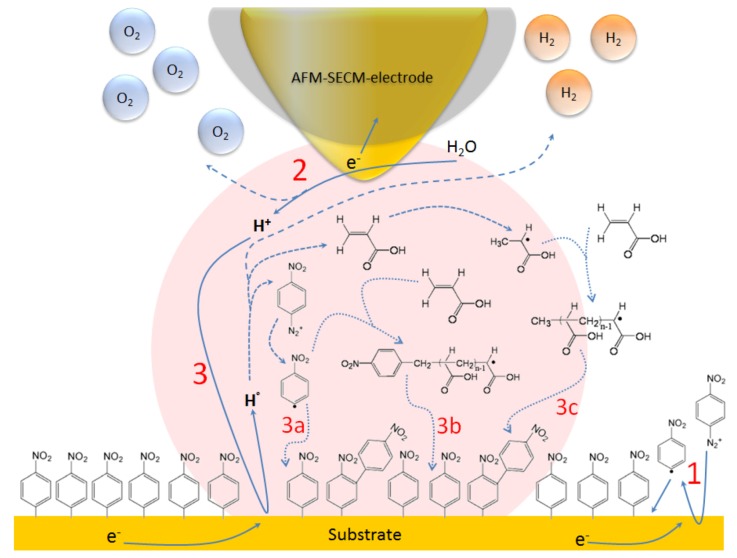
A simplified schematic representation of chemical and electrochemical reactions leading to the line grafting. (**1**) Reduction of the diazonium salt on the substrate to form a polynitrophenylene-like film; (**2**) Water oxidation at the AFM-SECM-electrode; (**3**) Concomitant reduction of protons on the substrate and formation of a localized grafted coating onto the top of the primer polynitrophenylene (PNP) film by radical reaction. (**3a**) Formation of a phenyl radical and reaction with PNP film; (**3b**) Phenyl radical initiates the first vinylic (plausible) radical polymerization. Reaction of the formed macroradicals with the grafted layer; (**3c**) Formation of radical monomer, initiation of the second (plausible) vinylic radical polymerization and reaction of the formed macroradicals with the grafted layer.

On the basis of recent experimental results [33,34], it clearly appears that localization is mainly controlled by the spatial extension of the current lines between the working and the counter-electrodes (see Figure 7). In fact, these current lines have the same distribution as those of the electric field that would exist between the two electrodes in a perfect dielectric medium. Indeed, the local nano-electrografting is based on the local generation of free radicals confined in the gap between the apex and the substrate. However, it is important to notice that the electrochemical environment is very complex, and it is really difficult to evaluate precisely the role played by each component of the electrolytic solution on the localized process. We are thus reduced to express reasonable assumptions to explain the localized process. It is proven that cathodic electrografting of vinylic monomers on conducting surfaces can be achieved under protic conditions, provided the polymerization is radically initiated by an electro-active moiety. In fact, as shown in Figure 8, the cathodic current produces nitrophenyl radicals from nitrobenzenediazonium tetrafluoroborate in the vicinity of the cathode, which immediately graft onto the electrode surface and generate a thin polynitrophenylene (PNP) film. Moreover, the narrow working distance limits the diffusion time of the radical species generated at the working electrode surface and ensures a small reaction area. Hence, the vinylic monomers in the electrolyte solution may play the role of trappers, which also limit free-radical diffusion and initiate a vinylic radical polymerization. This phenomenon is very similar to the well-known “chemical lens” phenomenon [35], where the diffusion of the electrodes generated species and that of the scavengers are in an opposite direction. In fact, when the chemical reaction between the trapper and the generated species is sufficiently fast, the surface modification of the substrate will be spatially limited to a thin zone surrounding the tip where the two species meet. Moreover, as shown in Figure 8, the described electrochemical system works as a “tip generator/substrate collector” system. It is important to remember that proton reduction plays a central role in polymer layer growth [36]. The major part of the hydrogen radicals gives gaseous hydrogen, but a very small fraction may initiate radical polymerization. According to the Osteryoung *et al*. [37,38] model, in the absence of a supporting electrolyte, protons migrate rather than diffuse from the tip to the substrate. It is well known that the migration effect is only sensitive to the current lines (see Figure 7), which then probably leads to a well-defined grafting localization. At the end of the propagation reaction, macroradicals react with the previously grafted PNP layer to form the final grafted film. AFM topographical characterization showed that the film thickness of the final grafted lines is about 40 nm. The nanometric thickness could be related to the flux convection profile of the solution dependence on the rate at which the probe is moved [39]. Moreover, recent SECM experimental works with different ultramicroelectrodes (UME) sizes showed that the grafted lines thickness follows an exponential decay, as a function of the scan rate. In fact, the coating thickness depends on the time spent over one point (tip speed). These phenomena could explain, by analogy, the 40 nm thickness of the coating, which is certainly related to the 18 µm s^−1^ AFM-SECM-electrode velocity.

## 4. Conclusions

In conclusion, these results demonstrate for the first time the feasibility of combined AFM-SECM technology for an electrochemical use on agarose. Moreover, the large capabilities of this technique were proven by the local nano-electrografting process. Experimental investigations show that the homemade AFM-SECM-electrode apex dimension, the short working distance and, particularly, the electrolysis of water are the key factors responsible for the nano-electrografting.

A detailed description of the information about the technical reproducing of AFM-SECM experiments and the manufacturing of homemade electrodes was reported. Indeed, the development of AFM-SECM-electrodes represents a significant advancement in SPM methodology and is a first step towards improved nano-processes for the nano-electrografting. In fact, a better experimental control and an AFM-SECM-electrode improvement (more rigid and stiffer) will allow for making more complex and clearer nano-patterns on surfaces.
